# Biological evolution and human cognition are analogous information processing systems

**DOI:** 10.3389/fpsyg.2023.1330345

**Published:** 2024-01-05

**Authors:** Juan C. Castro-Alonso, Alejandro A. Hidalgo, John Sweller

**Affiliations:** ^1^School of Education, University of Birmingham, Birmingham, United Kingdom; ^2^School of Pharmacy, Universidad Andres Bello, Santiago, Chile; ^3^School of Education, University of New South Wales, Sydney, NSW, Australia

**Keywords:** evolution by natural selection, genetic and epigenetic systems, human cognition and cognitive architecture, long-term memory and working memory, cognitive load theory

## Abstract

The mechanisms that govern biological evolution and human cognition are analogous, as both follow the same principles of natural information processing systems. In this article, we describe the following five principles that provide an analogy between biological evolution and human cognition: (a) Randomness as Genesis Principle and (b) Borrowing and Reorganizing Principle, which indicate how natural information processing systems obtain information; (c) Narrow Limits of Change Principle and (d) Information Store Principle, which indicate how information is processed and stored; and (e) Environmental Organizing and Linking Principle, which indicate how stored information is used to generate actions appropriate to an environment. In human cognition, these analogs only apply to cognitive processes associated with biologically secondary knowledge, the knowledge typically taught in educational institutions. Based on these five principles, cognitive load theory researchers have provided diverse prescriptions to optimize instructional activities and materials. We conclude by discussing general instructional implications and future research directions based on this analogy.

## 1 Introduction

Evolution by natural selection is traditionally seen as the biological theory that explains the diversity of all living forms, ranging from unicellular microorganisms, such as bacteria, to ecological populations of diverse multicellular organisms such as plants and animals. Indeed, evolution by natural selection is usually regarded as the central theory of biology (see Urry et al., 2021).

Despite its importance as a biological theory, evolutionary theory can also provide a template to explain processes in other disciplines. Simonton (1999) suggested that evolutionary theory can explain all historical and developmental phenomena that randomly generate alternatives to be selected, preserved, and reproduced (see Campbell, 1960; Gabora, 2019). Human cognition provides an example of such a system. Hence, as biological evolution is a well-established and developed theory, it can provide a rich source analogy for human cognition (Pringle, 1951; Sweller and Sweller, 2006; Paas and Sweller, 2022).

The suggestion that biological evolution is analogous to human cognition, learning, or culture is not new (Pringle, 1951; Campbell, 1960; Popper, 1979; Mesoudi et al., 2004; Reisman, 2013; Kouvaris et al., 2017) and can even be traced back to Darwin (1871/2003). More recent literature considers that biological evolution and human cognition are two examples of *natural information processing systems* (Sweller, 2003, 2020, 2022; Sweller and Sweller, 2006; Sweller et al., 2011; Paas and Sweller, 2022).

Nevertheless, while these analogies are well-known, they have had limited influence, possibly because evolutionary psychology is a relatively new discipline, with evolutionary educational psychology even more recent (see Geary, 2008, 2012). The aim of the present theoretical article is to describe further links between some aspects of biological evolution and human cognition, in the understanding that analogies are powerful tools that aid in aligning, comparing, and transforming information between different sources (see Richland and Simms, 2015; Petchey et al., 2023). By fostering connections between these theories, we can expect contributions to research on human cognition. Evolutionary theory with its close connections to genetics provides a far older, more detailed science than cognitive science and instructional design. Identifying analogical information processing structures can allow the application of well-established evolutionary principles to cognitive processes that lead to new insights concerning those processes.

## 2 Natural information processing systems

As described by Sweller and Sweller (2006), five common principles are shared by evolutionary and cognitive processes. Parallel descriptions can be given of the five principles from evolutionary and cognitive perspectives (see Table 1; Sweller, 2020, 2022).

**Table 1:** Natural information processing system principles.

Principle	Function	Evolution	Cognition
Randomness as genesis	Acquire information	Genomic sequences are generated	Information is generated
Borrowing and reorganizing	Acquire information	Genomic sequences are borrowed from other genomes	Information is borrowed from other people
Narrow limits of change	Manage information	Genomic sequences are limited by the epigenetic system	Information processing is limited by working memory
Information store	Store information	Genomic sequences are stored in the genome	Information is stored in long-term memory
Environmental organizing and linking	Employ stored information to govern action	Genomic sequences are activated or deactivated by the epigenetic system based on environmental signals	Information relevant to the extant environment is transferred from long-term memory to working memory to govern action

Adapted from Sweller and Sweller (2006).

### 2.1 Randomness as Genesis Principle

Initially, information must be generated or created. Evolution and cognition are natural systems with creative functions (see Campbell, 1960). The Randomness as Genesis Principle and the following principle (the Borrowing and Reorganizing Principle) provide the machinery by which natural information processing systems acquire information.

#### 2.1.1 Evolution: generating novel genomic sequences

Evolution has two different ways to cope with environmental information. If the environment is predictable and stable, then the Information Store Principle and the Environmental Organizing and Linking Principle are used. These principles will be discussed below. In contrast, if the environment is less stable, evolution must provide the more creative method of *generate and test*. The variability generator for evolution is *mutation*, which is a change in the genome, specifically in its DNA sequences (for a classic review, see Drake, 1969).

While genomic mutation provides the ultimate generating engine for the novelty and creativity of evolution, in isolation it is useless. Mutation is only valuable if it precedes a test of effectiveness. Many random mutations will have either no function or a negative outcome (Drake, 1969). Mutations with a negative outcome must be eliminated.

**Figure 1:**
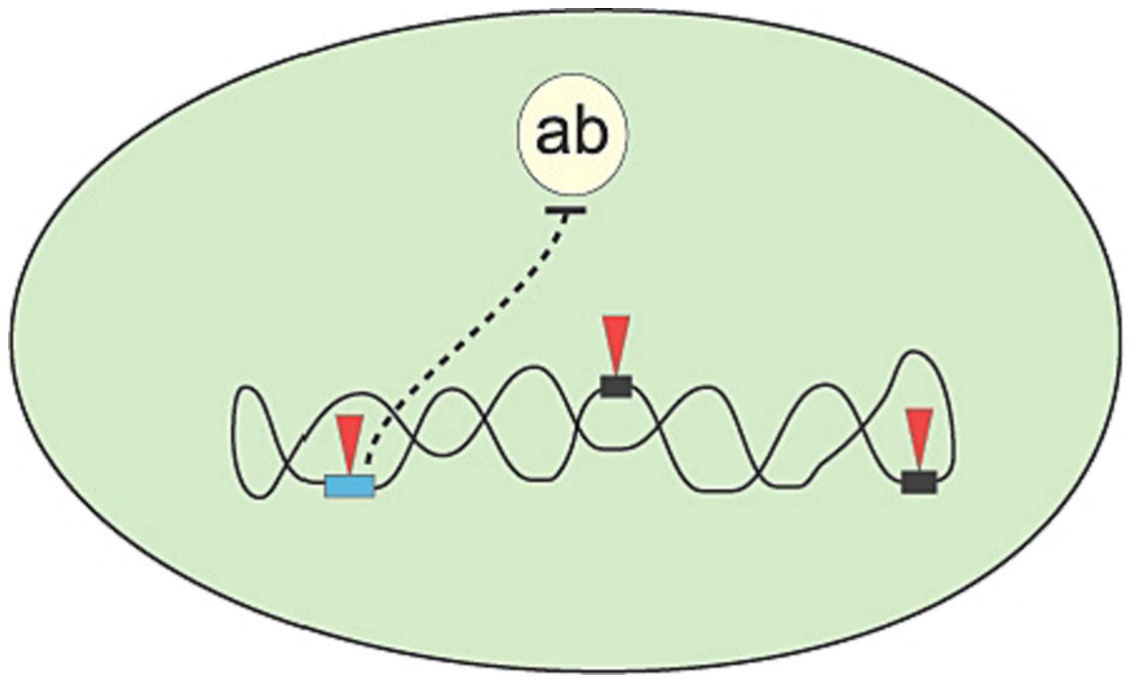
The bacterium (large oval) is challenged with a new antibiotic (ab), for which it has no defense genes to nullify its killing action. However, by accumulation of point mutations in gene sequences (arrow heads pointing to rectangles), the bacterium might develop a new gene (light rectangle on the left) coding for a function that will neutralize the harmful action of the antibiotic.

In this manner, a random change is tested for effectiveness, and only effective changes are retained. To sum up, the mechanisms of evolution follow the logic inherent in generate and test: Mutations generate novel structures that are subsequently tested for effectiveness. For example (see Figure 1), mutations may aid in the survival of a simple organism (bacterium) when it faces potential death due to an antibiotic.

#### 2.1.2 Cognition: generating novel information

As in evolution, when problem solvers determine their next move, they can use two separate processes (Sweller, 2009). First, if the current problem state is familiar to them and has a known next step or move, the Information Store Principle and the Environmental Organizing and Linking Principle are relevant (see Sweller, 1989) and will be discussed below.

Second, when problem solvers are challenged with an unfamiliar or novel problem, by definition, one or more of the steps or moves cannot be completely determined by retrieval from their stores of information (long-term memory). Problem solvers facing a novel problem do not have all the prior knowledge that could direct and assist them, although they usually have some helpful knowledge (see Perry et al., 2021), such as being able to read the problem. In these scenarios, instead of retrieving information from long-term memory, solvers use a generate and test procedure, processed in working memory. This is analogous to the generate and test of mutations in evolution.

Typically, the procedure of generate and test in cognition is part of a means-ends problem solving strategy that involves considering the current problem state, considering the goal state, finding differences between them and finding a move that will reduce those differences (Newell and Simon, 1972; Novick and Bassok, 2005). This means-ends strategy is common when problems are closed, in the sense that they have few possible steps or moves. The problem solver must discriminate from these possible available steps or moves that might decrease the gap between a current problem state and the goal state. If there is existing knowledge in long-term memory, the best move can be chosen.

In contrast, if knowledge in long-term memory is not available, the alternative option is to employ a random generate and test procedure. Note that this is a simplification, as some partial knowledge or mental structure to assist in solving a problem will always be available (see Perry et al., 2021). In this case, this partial knowledge may be used to predispose the problem solver toward particular moves, while pure random choice is only used when there is no available information to assist in choosing between moves. Once a move is chosen, it can be tested for effectiveness. This process can be considered an “invention” (Perry et al., 2021). If the move decreases gaps between the current problem state and the goal state, it is accepted, a new current state is arrived at, and the process can be reiterated.

Successful moves can be stored for future use in long-term memory (Sweller and Sweller, 2006). This trial-and-error tactic is inefficient for acquiring knowledge, compared to obtaining it from another person (see below Borrowing and Reorganizing Principle; see also Sweller, 2015). Danchin et al. (2004) also differentiated between these two tactics, which they termed as acquiring *personal information* or *social information*. They described the latter as leading to more accurate predictions to solve problems.

### 2.2 Borrowing and Reorganizing Principle

This principle is the second principle allowing natural information processing systems to acquire information. While randomness as genesis provides the initial creative engine generating novel information, natural information processing systems obtain most of their information via the Borrowing and Reorganizing Principle. Information characteristically is acquired by borrowing from other stores of information rather than being generated by the Randomness as Genesis Principle.

#### 2.2.1 Evolution: acquiring information from other genomes

Borrowing information is a central mechanism in evolution. Almost the entire genome of organisms is borrowed from the genomes of ancestors, allowing the accumulated advantages of a particular genome to be retained indefinitely. Sexual and asexual reproduction permit this advantage to be transmitted despite the characteristically immense size of typical genomes. In this manner, the large amount of information required by a genome can be acquired quickly and efficiently. Thus, the *borrowing* component of this principle is highly efficient.

We can use simple organisms, such as bacteria, to describe multiple ways in which they transfer information between each other, not only as a means of reproduction. One relatively direct way of transference is called *genetic transformation* (see classical experiments by Avery et al., 1944; Matthey and Blokesch, 2016; Dubnau and Blokesch, 2019), where a bacterium can incorporate genetic material encountered in the environment, usually from other bacteria. Bacteria can also incorporate external genetic information via a less direct method, *transduction*, where a bacteria-infecting virus acts as carrier of genetic information (Calero-Cáceres et al., 2019). Another rather indirect way is by *conjugation*, where genetic information is transferred though a sexual structure (for a review, see Lanka and Wilkins, 1995). A relatively newly discovered indirect process, which is *vesicle-mediated*, uses cellular membrane vesicles that fuse two membranes to transport genetic information from donor to receptor bacterium (see Domingues and Nielsen, 2017).

Millanao et al. (2021) reviewed these mechanisms when used by bacteria to resist the negative action of antibiotics. The outcome of all these transfer processes is to reorganize the information in the host genome before being tested for effectiveness. This is the *reorganizing* component of the principle, which recognizes that the information borrowed is commonly altered by the recipient. Only effective reorganizations that increase the chances of survival are retained in the genome and transferred to the next generation (Sweller and Sweller, 2006).

#### 2.2.2 Cognition: acquiring information from other people

Commonly when learning, we borrow information from other human beings, listening to what they say, imitating what they do (see Bandura, 1986), or reading what they write (see Heyes, 2012; Gabora, 2019). Analogously to the processes of evolution, cognition has multiple ways to transfer information between individuals. An example of typical borrowing in the classroom occurs when students learn from an instructor modeling a procedure. Another typical example of borrowing occurs when students learn from reading a text written by an instructor.

As in evolution, the borrowing component of this principle in cognition is efficient. We can accumulate large amounts of information far more quickly by borrowing it from others than by any other means. Indeed, it would be impossible for us to acquire the massive amounts of information acquired in education and training contexts other than by borrowing it from others (see also Danchin et al., 2004). This implies that the most effective instructional method uses direct and explicit teaching, which allows information to be borrowed from a more knowledgeable source, as opposed to instructional methods using minimal guidance (Sweller, 2015). As has been indicated previously (Mayer, 2004; Kirschner et al., 2006; Chen et al., 2018b), minimal guidance during learning has provided little evidence of effectiveness.

As in evolution, the information borrowed is also reorganized by the recipient. The borrowed information is combined with old information previously stored, and this combined information has been termed *schemas* (see Rumelhart, 1981; Shing and Brod, 2016) or *chunks* (see Cowan, 2001). When learning, those features of the instructional information that conform with previously stored schemas, are refined or emphasized; at the same time, novel information that does not match information stored in long term memory is smoothed (Bartlett, 1932; Thorndyke and Hayes-Roth, 1979). Therefore, the same information is reorganized in a different manner depending on previous knowledge.

### 2.3 Narrow Limits of Change Principle

The Narrow Limits of Change Principle and the Information Store Principle (see below) indicate how natural information processing systems manage and store information. There is a structural effect that flows from the initial generation of novel information when using the Randomness as Genesis Principle. As explained by Sweller and Sweller (2006), an information processing system that generates combinations of elements in a random way runs the risk of large combinatorial explosions. For example, there are 3! = 6 permutations of 3 elements, but there are 10! = 3,628,800 permutations of 10 elements. In consequence, it may be simple for biological organisms to use generate and test when the options are only six possibilities. Choosing from more than three million possibilities will be much more difficult and take much longer.

For this reason, natural information processing systems require a mechanism to safeguard that the number of combinations to be tested is kept to manageable numbers. In the case of genetics or evolution, the epigenetic system plays this role. In the case of human cognition, the limits of working memory play a similar role.

#### 2.3.1 Evolution: epigenetic system

The store of genetic information (genome) of prokaryotes and eukaryotes is composed of double stranded DNA, with information in one strand being complementary to that of the opposite strand. This duplicated property of DNA is fundamental for its effective replication. In fact, DNA replication is almost a perfect process, with only one error per about ten million nucleotides (the units or structural monomers that form DNA) during replication (see Bird, 2007). The rate of these errors (mutations) is kept low by different mechanisms (Bjedov et al., 2003) coordinated by the epigenetic system.

The *epigenetic system* comprises all mechanisms that control how the genetic information in the genome is expressed as useful molecules for the organisms (e.g., proteins). Note that “epigenetic” is normally used in a narrower sense, for example, when molecular biologists describe information that is inherited by the offspring not via genes in the genome (see Haig, 2004; Bird, 2007; Mesoudi et al., 2013). In bacteria, these microorganisms, under some conditions, use epigenetic inheritance (not gene inheritance) to produce sub colonies with different characteristics, leading to a fast adaptation to their environment (see Veening et al., 2008). However, in this article we use epigenetic in a broader sense, as used by functional morphologists (see Haig, 2004), and we include both the heritable but also the most common non-inherited mechanisms that control the genetic expression of the information in the genome.

That the epigenetic system controls which genes are exposed to be expressed and which are inhibited leads to two consequences: (a) the *reduction* of overall genes being expressed, which also reduces the possibilities of random mutations and errors in the genome (Sweller and Sweller, 2006); and (b) the *selection* of genes that will be expressed, which is also a way of allowing only these DNA segments to risk developing mutations (see Molan and Žgur Bertok, 2022).

Regarding the first consequence, which is the reduction of overall genes being expressed, bacteria and more advanced cells pack the DNA as *chromatin* by employing histone proteins, in order to inhibit and protect the genomic information (for a review, see Weintraub, 1985; Zhang and Meaney, 2010). There are specific enzymes that unpack these tight bonds between histones and DNA, allowing the genome to express its information; there are also enzymes that repack these links and inhibit genome expression (Zhang and Meaney, 2010). In addition to these mechanisms that use proteins binding the DNA, there are direct DNA chemical modifications on the chromatin (e.g., methylation) that can silence the expression of that genomic information (Zhang and Meaney, 2010). Binding of histones, DNA methylation, and similar chromatin regulations warrant that the number of mutations in the genome is not enormous and thus potentially harmful for the organisms.

Concerning the second consequence, which is the selection of genes being expressed, the epigenetic system determines where mutations are more likely to occur. Although mutations are to a major extent randomly situated in the genome, there are more probable sites (*hotspots*) for mutations to occur (Coulondre et al., 1978; Wright, 2000; Chan and Gordenin, 2015). Opportunistic mobile genetic elements, such as transforming genes from nearby bacteria (see Dubnau and Blokesch, 2019) or genetic information in vehicles such as viruses and transposons (see Fricker and Peters, 2014), normally use these spots to produce mutations.

#### 2.3.2 Cognition: working memory

Working memory is limited (see Oberauer et al., 2018), both in capacity (Miller, 1956; Cowan, 2001) and in duration (Peterson and Peterson, 1959). We can hold no more than about seven elements of information in working memory and can process no more than about three to four. Although working memory is limited, its limitations are not a disadvantage, but are advantageous and central to the mechanisms of natural information systems (Krems and Wilkes, 2019). There are two reasons why working memory should be limited when dealing with information acquired via the Randomness as Genesis Principle or the Borrowing and Reorganizing Principle.

The first reason, as explained above, concerns random generation of problem solving moves. When dealing with novel information, we cannot know how elements should be organized. We must handle that information using the random generate and test procedure that is central to the Randomness as Genesis Principle. Random generate and test can be functional when dealing with only a few elements at a time. It cannot function when dealing with more than 3–4 elements simultaneously (Cowan, 2001). Working memory determines which elements will be randomly generated and tested, analogously to the epigenetic system in biological organisms.

The second reason concerns the size of changes to a permanent store of information. The limits of working memory guarantee that a large quantity of information is never processed simultaneously. Since only a small quantity of information can be handled by a limited working memory, any changes to the long-term memory store (see below) are limited, thus lowering the risk of damaging the knowledge structures that have been successfully acquired over extensive periods (Sweller, 2010). Analogously, as described above, genomes do not rapidly change. The Narrow Limits of Change Principle ensures that changes are small and incremental.

### 2.4 Information Store Principle

The Information Store Principle is the second principle concerned with the manner in which information, once acquired, is managed. To deal with a complex, constantly changing environment, natural information processing systems require a very large store of information. Genomes provide that store in the case of cellular evolution, while long-term memory has an analogous role in human cognition.

#### 2.4.1 Evolution: genome

The genome of each living species is its store of genetic information to deal with a complex environment that constantly changes. The information is stored in the genome as the combination of monomers (nucleotides) that constitute the sequences of DNA. That information held in a genome is substantial, so the species can manage to thrive, in spite of variable environmental conditions (Sweller et al., 2011).

In prokaryotes (bacteria), those that live in more stable environments (e.g., inside cells of the human body), tend to have smaller genomes than bacteria living in more fluctuating environments (e.g., soil; see Bentley and Parkhill, 2004). This differential size of genomes occurs because larger stores of information are needed in organisms that cope with more variable than less variable environments. However, even the most basic known species of bacteria that live in stable environments (e.g., the human intracellular bacterium *Mycoplasma genitalium*), contain large stores of information, sufficient to make approximately 200–500 different genomic products, mostly proteins (Glass et al., 2006; Moran and Bennett, 2014).

**Figure 2:**
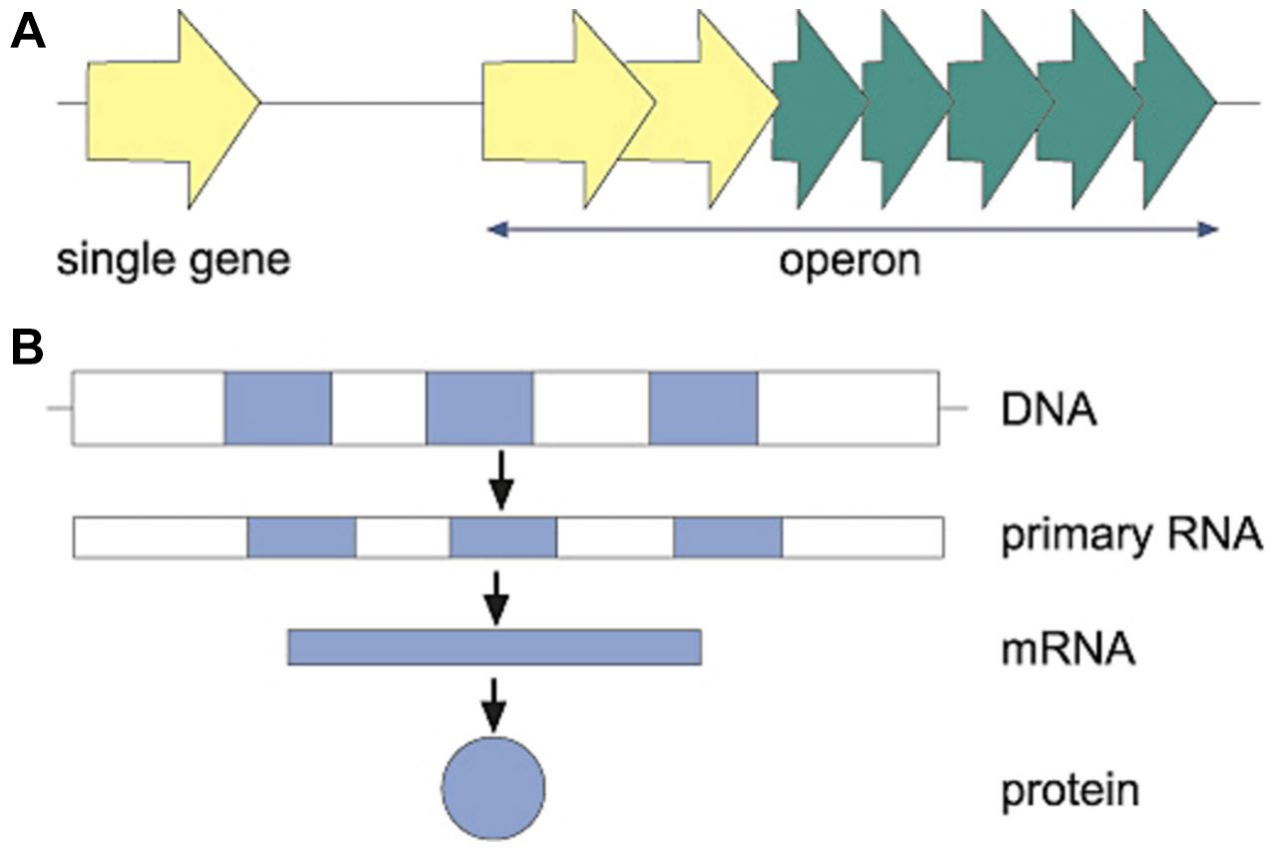
**(A)** In prokaryotes (bacteria), the genetic information can be organized as either single genes (light arrow) or as clusters of genes, operons (light and dark arrows), which are expressed together. This organization as operons is part of the economy of prokaryotes, as their genomes are smaller than those of eukaryotes. The organization in operons allows a prompt response when various protein products are needed in a changing environment. **(B)** In eukaryotes, with larger genomic information than prokaryotes, there are both exons (dark) and interspaced introns (white). Only the information in the exons constitute the mRNA and final proteins.

In bacteria (see Figure 2A), the genetic information can be read from either single genes or clusters of genes organized in *operons* (see Lawrence, 2002). In species more complex than bacteria, such as eukaryotic cells, operons have not been reported and the DNA is organized in a unique way that has not been observed in bacteria. As such, in eukaryotes, the nuclear genome includes non-coding DNA information (*introns*, which do not form genomic products) as well as coding DNA sequences (*exons*, which are translated into proteins and other genomic products), all in the nucleus of the cell (see Figure 2B). Moreover, the genome of the eukaryotic cell includes non-nuclear information (e.g., from mitochondria or chloroplasts; see Noguera-Solano et al., 2013). Producing proteins based on different exons and introns is a way to control gene expression following environmental cues, as described in the next principle.

#### 2.4.2 Cognition: long-term memory

Analogously to the genome, long-term memory can contain a vast quantity of information. For example, expert chess players can store approximately 10,000 different game patterns as schemas or chunks (Simon and Gilmartin, 1973). Similarly, all humans have huge quantities of information in their long-term memory stores in order to assimilate the plethora of information required to function in modern societies (Sweller and Sweller, 2006).

The role of long-term memory in problem solving first became apparent from work on the game of chess. De Groot (1965) initiated this line of research, first published in the 1940’s. He showed chess masters and less able players a chessboard pattern of pieces copied from a real game for 5 s, and then removed the chessboard and asked the players to reproduce the pattern from memory. It was observed that chess masters were dramatically better than the less able players at this task.

In a subsequent study, Chase and Simon (1973) observed that this difference disappeared when random chessboard patterns were used, with both masters and less able players equally poor at remembering the configurations (see also Reingold et al., 2001). In other words, chess masters only performed well on a chessboard pattern memory task when they were faced with configurations that had occurred in real games, and that they had studied. These results suggested that, after extensive practice, chess masters had stored in long-term memory many thousands of chessboard configurations with their best associated moves.

The same process differentiates novices from experts in all subject areas of human expertise (see Ericsson and Charness, 1994). The review by Novick and Bassok (2005) showed this novice–expert difference in disciplines such as physics, mathematics, geometry, and biology. Similarly, the meta-analysis by Sala and Gobet (2017)—which considered, among others, the skills of computer programming, bridge, chess, and music—reported similar differences. In short, experts have a huge quantity of domain-specific information about their area of expertise stored in long-term memory (see also Sweller, 2015).

### 2.5 Environmental Organizing and Linking Principle

Once information is acquired and managed, natural information processing systems must employ the information to govern the moves or actions that are appropriate for a given environment. Based on the Environmental Organizing and Linking Principle, a natural information processing system gathers environmental cues to allow stored information to determine appropriate responses. The epigenetic system coordinating genomic expression, and working memory and long-term memory coordinating cognition, provide the conduits.

#### 2.5.1 Evolution: epigenetic system

Environmental signals indicate, via the epigenetic system, which stored information should be *activated* and which information should be r*epressed* (or remain repressed). While information stored in the genome determines the *potential* activities that the cells can perform, the *actual* activities are determined by the control of the epigenetic system.

We consider three mechanisms in which this system can control gene expression following environmental cues: (a) transcription factors, (b) chromatin or DNA packaging, and (c) exons versus introns. Regarding the first two mechanisms, transcription factors and packaged DNA are common to all cells, including bacteria and more advanced organisms. The presence of exons and introns is only observed in the complex eukaryotic cells, and not in the cells of bacteria (prokaryotes).

Regarding the first mechanism, transcription factors are large complexes that regulate the activation or repression of genes depending on the presence or absence of specific molecules in the environment (Gilbert and Müller-Hill, 1966). These regulatory complexes respond rapidly to environmental cues and are the common way of turning genes on or off employed by the cells (see Zhang and Meaney, 2010). In a sense, using transcription factors is the fine tuning of the slower effects produced by the second mechanism, which involves the more permanent regulation of genomic expression by packed chromatin, histones, and DNA methylation. Although chromatin regulations tend to be slower than the effects of transcription factors, there is evidence that changes in chromatin can sometimes respond dynamically to environmental cues (Miller and Sweatt, 2007).

Exons and introns, which occur, for example, in plants and animals, provide a third mechanism for controlling gene expression by following environmental signals. In these evolved organisms, only exons contain genomic information that will lead to a product used by the cells (e.g., protein). The introns are the interspaced DNA sequences between the exons (see Figure 2B). Introns are more than eight times larger and mutate at a much higher rate than the exons (see Hall, 2012). *Alternative splicing* is the process by which the regulation of exons and introns follows cues from the environment. Alternative splicing results in a large genomic sequence that follows environmental cues to determine which of its including exons will be expressed, and thus which protein will be produced (see Green, 1991).

#### 2.5.2 Cognition: working memory and long-term memory

Analogously to the epigenetic system, stimuli from the environment trigger working memory to search for relevant information in long-term memory, in order to determine suitable actions. There are two important implications (Sweller and Sweller, 2006; Sweller et al., 2011). The first implication is that when working memory obtains information from long-term memory, the limits of working memory are overcome, as the information is effectively managed as a single element. Hence, working memory is limited in capacity and duration when it processes new information from the environment via the Narrow Limits of Change Principle, but these limitations do not apply when working memory is employed to retrieve information from long-term memory via the Environmental Organizing and Linking Principle. Once information is stored as schemas or chunks in long-term memory, indefinite quantities of that information can be conveyed to working memory for indefinite time periods (see Ericsson et al., 1993; Ericsson and Charness, 1994; Ericsson and Kintsch, 1995; Oberauer et al., 2018).

The second implication is that some information is activated by the environment via working memory, whereas other information remains inactive in long-term memory. For example, specific goals to achieve a task activate the relevant information stored in long-term memory, including knowledge, beliefs, values, and interests (see Doebel, 2020). Thus, for human cognition, cues from the environment indicate which information held in long-term memory should be conveyed to working memory to generate activity (see Shing and Brod, 2016).

## 3 Categories of information

With respect to cognition, the above principles provide a cognitive architecture that indicates how humans learn, think, and solve problems. It needs to be noted that this cognitive architecture does not apply to all categories of information with which humans must deal. Geary (1995, 2007, 2008, 2012) distinguishes between *biologically primary* and *biologically secondary* information. Humans have specifically evolved to unconsciously acquire primary information, so it is unnecessary to teach it. Examples of primary tasks are object manipulations (Castro-Alonso et al., 2015; Bokosmaty et al., 2017) and gestures (Zhang et al., 2022). The five principles discussed here do not apply when acquiring primary information. Rather, the cognitive architecture described by these five principles applies to the acquisition of biologically secondary information.

Biologically secondary information is information that we have not specifically evolved to acquire. It is information that we need for cultural reasons. Almost everything taught in training and education institutions (e.g., schools and universities) is biologically secondary, such as learning mathematics or reading. As described by *cognitive load theory* (see next section), the five principles apply to this secondary information that we acquire in formal institutions.

Also, most biologically primary information consists of generic-cognitive knowledge and skills associated with general learning and thinking while most biologically secondary knowledge and skills consist of domain-specific knowledge (Tricot and Sweller, 2014; Paas and Sweller, 2022). We do not need someone to teach us general problem solving strategies such as means-ends analysis (Kirschner et al., 2006; Sweller, 2023), but we do need to be taught domain-specific skills such as how to balance the two sides of an algebraic equation. Explicit instruction tends to be needed when acquiring domain-specific secondary information, unlike the acquisition of generic-cognitive primary information. Explicit instruction and the five principles are basic to cognitive load theory, as discussed next.

## 4 Cognitive load theory

Cognitive load theory is an instructional theory based on the above principles (see Sweller et al., 2011, 2019). Those principles constitute a cognitive architecture. The theory assumes that instruction is primarily aimed at fostering the acquisition of domain-specific, biologically secondary skills and knowledge.

The theory has provided a framework to devise instructional prescriptions based on instructional effects, each of which has been tested using multiple, randomized, controlled trials (see Sweller et al., 2011, 2019; Castro-Alonso et al., 2019a, 2021; Sweller, 2020, 2023; Hanham et al., 2023). Examples of the use of the instructional effects prescribed by cognitive load theory may be found in Garnett (2020), and Lovell (2020).

The instructional prescriptions (or *effects*) investigated using cognitive load theory are connected to the five principles described here. While there are many such effects (see Sweller et al., 2019), the *worked example effect* is one of the most commonly cited and will be discussed here. It occurs when students given worked examples to study obtain higher subsequent test scores than students given the equivalent problems to solve.

The above five principles explain why. In accord with the Randomness as Genesis Principle, domain-specific, biologically secondary information can be acquired slowly and inefficiently during problem solving but much more rapidly and efficiently from other humans via the Borrowing and Reorganizing Principle. Problem solving employs the Randomness as Genesis Principle, while studying worked examples uses the more efficient Borrowing and Reorganizing Principle, partly explaining the worked example effect.

Instruction usually deals with novel information, so both the above principles are concerned with the processing of novel information. The Narrow Limits of Change Principle indicates the characteristics of working memory when dealing with such novel information. Studying worked examples requires far less information to be processed by the limited capacity and duration working memory than solving the equivalent problems. As indicated above, solving a problem using means-ends analysis requires learners to simultaneously consider their current problem state, the goal state, differences between the states, and problem-solving operators that might reduce those differences (Newell and Simon, 1972; Novick and Bassok, 2005). A worked example eliminates those processes and so reduces working memory load.

Once novel information is processed by the limited capacity and duration working memory, unlimited amounts can be stored indefinitely as knowledge in long-term memory using the Information Store Principle. A worked example that properly organizes problem solving steps facilitates the storage of the problem solution in long-term memory.

Lastly, once information is stored in long-term memory, it can be retrieved back to working memory, following signals from the environment, to generate appropriate action in accord with the Environmental Organizing and Linking Principle. Solution information from a worked example can be readily used to efficiently solve problems by allowing problem solvers to quickly recognize a problem as belonging to a category for which the problem solver has a complete solution.

Based on this theoretical foundation, cognitive load theory assumes that the purpose of instruction is to allow learners to accumulate knowledge in long-term memory for later use (see also Paas and Ayres, 2014). All of the cognitive load theory effects, including the worked example effect, have this function.

## 5 Discussion

In the present article, we have provided five principles of natural information processing systems that apply to both biological evolution and human cognition, which consequently, are analogous. Concerning human cognition, the cognitive architecture mechanisms that flow from the five principles only apply to biologically secondary knowledge, such as learning to solve specific mathematics problems or write essays, but not to biologically primary knowledge (see Geary, 2012). In other words, the analogies that can be applied to human cognition from the evolutionary mechanisms presented, most commonly apply to the knowledge typically taught in formal institutions (e.g., schools and universities). As indicated above, cognitive load theory (see Sweller et al., 2011, 2019; Castro-Alonso et al., 2019a, 2021; Garnett, 2020; Lovell, 2020; Sweller, 2020, 2023) has applied these principles of natural information systems to investigate and produce instructional prescriptions aimed at fostering effective learning of biologically secondary knowledge.

### 5.1 Implications for instructional practice

A first general instructional implication of this article flows from the first two principles and has been supported by cognitive load theory research. As it is more efficient for us to obtain information from others (Borrowing and Reorganizing Principle) than from generating and testing it by ourselves (Randomness as Genesis Principle), teachers and instructors should promote direct and explicit instruction over unguided discovery learning (see Mayer, 2004; Kirschner et al., 2006; Chen et al., 2018b).

A second instructional implication flows from the third principle, and it has also been supported by cognitive load theorists. As the Narrow Limits of Change Principle operates on working memory, this memory processor cannot deal with large amounts of simultaneous novel information (see Sweller et al., 2019; Sweller, 2020). In consequence, teachers and instructors should follow the strategies of cognitive load theory (labeled “cognitive load theory effects” in the literature) to avoid students’ working memory being overloaded when they process novel information intended for learning (see also Castro-Alonso et al., 2018; Chen et al., 2018a). As indicated above, all cognitive load theory effects are based on multiple, randomized, controlled studies with most such effects concerned with reducing unnecessary working memory load (Sweller et al., 2019; Sweller, 2020).

A third general instructional implication is based on the fourth and fifth principles, and has also been actively investigated by cognitive load theory researchers. According to the Information Store Principle, experts have a vast quantity of information in their areas of expertise stored in long-term memory (see Ericsson and Charness, 1994), which, according to the Environmental Organizing and Linking Principle, can be processed in working memory when the environmental situation requires an appropriate action (see Ericsson and Kintsch, 1995). When considering the Information Store Principle, the Environmental Organizing and Linking Principle, and the Narrow Limits of Change Principle, it can be observed that novices in a given area have a lower working memory capacity in that area than more knowledgeable students. Consequently, teachers and instructors should adapt their instructional materials and activities depending on the expertise of their students, which follows from the *expertise reversal effect* of cognitive load theory (see Kalyuga et al., 2003; Chen et al., 2017; Castro-Alonso et al., 2021; Chen et al., 2023).

### 5.2 Future research directions

There are several suggestions for future research on human cognition based on the analogy with evolution. For example, regarding the Borrowing and Reorganizing Principle, there are less direct mechanisms (e.g., transduction, which uses a carrier virus) and more direct mechanisms (e.g., genetic transformation, which does not use intermediary carriers) to transfer information to the genome. Analogously, there might also be indirect and more direct mechanisms to transfer information to long-term memory, such as those using or not using an intermediary sensory memory (*cf.* Malmberg et al., 2019). Concerning the Environmental Organizing and Linking Principle, it would be interesting to investigate the cognitive analogs of the phenomena of selecting which genes to express and which to repress. In other words, how does working memory select appropriate information from long-term memory and why does it sometimes select the wrong information resulting in the *Einstellung* effect (see Luchins, 1942)?

Another example of potential future research is related to the Information Store Principle. As some organisms have extra non-nuclear stores, it would be interesting to investigate if there are cognitive stores in addition to long-term memory. For example, *embodied cognition* investigates the simultaneous processing of brain, body, and environment (see Sepp et al., 2019; Castro-Alonso et al., 2019b), so some of these accounts could consider human limbs and their nervous system controls as additional stores that help cognitive processes.

Other similar analogies between cognition and evolution could be made. Also, the boundaries of cognition could be expanded by including, for example, metacognition (see Perry et al., 2019) or emotion (see Plass and Kalyuga, 2019). However, as cautioned by Oppenheimer (1956), all analogies, even if inspirational, must be empirically tested, to find their limits (*cf.* Petchey et al., 2023). In that sense, the proper scientific methods that cognitive load researchers have used in the development of the theory—randomized, controlled, and replicable experiments (Sweller et al., 2011, 2019)—should be used in the testing of novel instructional procedures inspired by this analogical approach.

## 6 Conclusion

Building on the five principles of natural information processing systems (Sweller and Sweller, 2006), in this theoretical article we have described analogies between evolution and human cognition for biologically secondary knowledge. We indicated that several links can be made between the genetic mechanisms of different organisms (e.g., bacteria) and the mechanisms of human cognitive architecture when dealing with secondary knowledge. This analogy is central to cognitive load theory. That theory has been used to generate novel instructional procedures which have been tested in a very large range of experiments using randomized, controlled designs. In turn, the results of that empirical work provide support for the theory.

## Data availability statement

The original contributions presented in the study are included in the article/supplementary material, further inquiries can be directed to the corresponding author.

## Author contributions

JC-A: Writing – original draft, Writing – review & editing. AH: Writing – review & editing. JS: Conceptualization, Writing – original draft, Writing – review & editing.
